# Examining the relationship between land use and childhood leukemia and lymphoma in Tehran

**DOI:** 10.1038/s41598-024-63309-z

**Published:** 2024-05-30

**Authors:** Samira Norzaee, Masud Yunesian, Arsalan Ghorbanian, Mahdi Farzadkia, Roshanak Rezaei Kalantary, Majid Kermani, Seyed Mohammad-Kazem Nourbakhsh, Aziz Eghbali

**Affiliations:** 1 Research Center for Environmental Health Technology, Iran University of Medical Sciences, Tehran, Iran; 2 Department of Environmental Health Engineering, School of Public Health, Iran University of Medical Sciences, Tehran, Iran; 3 Department of Environmental Health, School of Public Health, Tehran University of Medical Sciences, Tehran, Iran; 4 Center for Air Pollution Research, Institute of Environmental Research, Tehran University of Medical Sciences, Tehran University of Medical Sciences, Tehran, Iran; 5 Department of Photogrammetry and Remote Sensing, Faculty of Geodesy and Geomatics Engineering, K. N. Toosi University of Technology, Tehran, Iran; 6 Department of Pediatrics, Pediatric Hematology and Oncology Section, Imam Khomeini Hospital Complex, Tehran University of Medical Sciences, Tehran, Iran; 7 Pediatric Congenital Hematologic Disorders Research Center, Research Institute for Children Health, Shahid Beheshti University of Medical Sciences, Tehran, Iran

**Keywords:** Cancer, Environmental sciences

## Abstract

We conducted a hospital-based case–control study to explore the association between proximity to various land use types and childhood leukemia and lymphoma. This research involved 428 cases of childhood leukemia and lymphoma (2016–2021), along with a control group of 428 children aged 1–15 in Tehran. We analyzed the risk of childhood cancer associated with land use by employing logistic regression adjusted for confounding factors such as parental smoking and family history. The odds ratio (OR) for children with leukemia and lymphoma residing within 100 m of the nearest highway was 1.87 (95% CI = 1.00–3.49) and 1.71 (95% CI = 1.00–2.93), respectively, in comparison to those living at a distance of 1000 m or more from a highway. The OR for leukemia with exposure to petrol stations within 100 m was 2.15 (95% CI = 1.00–4.63), and for lymphoma it was 1.09 (95% CI = 0.47–2.50). A significant association was observed near power lines (OR = 3.05; 95% CI = 0.97–9.55) within < 100 m for leukemia. However, no significant association was observed between power lines and the incidence of childhood lymphoma. There was no association between bus stations, major road class 2, and the incidence of childhood leukemia and lymphoma. In conclusion, our results suggest a possible association between the incidence of childhood leukemia and proximity to different urban land uses (i.e., highways and petrol stations). This study is the first step in understanding how urban land use affects childhood leukemia and lymphoma in Tehran. However, comprehensive studies considering individual-level data and specific pollutants are essential for a more nuanced understanding of these associations.

## Introduction

Childhood cancer is a term used to describe cancer in children and adolescents^1^. Leukemia and lymphoma are two common types of blood cancer in children^2^. The two primary forms of leukemia in children are Acute Lymphoblastic Leukemia (ALL) and Acute Myeloid Leukemia (AML)^3^, and Hodgkin's (HL) and non-Hodgkin's lymphoma (NHL) are the two primary forms of lymphoma^4^. Studies in Iran show that the incidence and prevalence of leukemia and lymphoma have significantly increased over time^5^. A meta-analysis conducted in Iran reveals that the incidence rate of cancer in children aged 0 to 14 years was 16.80 per 100,000 for boys and 16.56 per 100,000 for girls^6^. Although the incidence of cancer in children in Iran is increasing, the available data and studies are limited.

There are only a limited number of known factors that can contribute to the development of childhood leukemia and lymphoma, including genetic syndromes such as Down syndrome, exposure to high levels of ionizing radiation, and having an abnormal birth weight (low birth weight and high birth weight). However, a significant portion of the cases lack a dominant genetic factor. The geographical variations of childhood cancer incidences indicate that environmental factors associated with geographical locations can also play a significant role in increasing or decreasing cancer incident rates. Despite extensive epidemiological studies, there is still a lack of information on the environmental factors affecting the incidence of childhood cancer. As a result, more epidemiological research is needed to determine the relationship between environmental factor exposure and childhood cancer incidence. According to studies, environmental pollutants emitted by traffic are associated with various types of cancer^7^. On the other hand, other than individual and social factors, land use could contribute to the incidence of cancer in children^8^. In reality, economic growth and the development of land use, such as the construction of highways, petrol stations, etc., are strongly correlated^9^. The consequences of land use developments and transitions not only have significant environmental implications but also have a close relationship with people's health^10^. Despite its importance, only a few studies have been carried out at regional levels concerning land use association with the incidence of cancer.

The capital and largest city of Iran is Tehran, which accommodates over 10% of the country's population. According to the 2016 census, 8.7 million people live in Tehran. Although the rapid economic growth of Tehran in the past three decades has brought many benefits, the adverse health effects of rapid urban development and industrialization pose an important challenge to public health^11,12^. The relationship between land use and cancer incidence needs to be investigated, given the increase in childhood cancers. Some evidence shows that the land use type in urban areas can directly affect the pollutants released into the environment and, ultimately, increase the incidence of various diseases^13^. However, given the limited research conducted and the existence of contradictory results in such studies, more investigation seems essential to clarify the influential factors in the incidence of childhood cancer. Unfortunately, there hasn't been much study on the effect of proximity to land use and the incidence of cancer, especially in children in Tehran. Therefore, the main purpose of this study was to investigate the relationship between proximity to land use of roads, petrol stations, power lines, and bus stations and the incidence of leukemia and lymphoma in children living in Tehran.

## Materials and methods

### The study area

Tehran is located in the northern part of Iran at coordinates 51°23′20″ E and 35°41′21″ N. The city encompasses an area of 676.31 km^2^ and has a population of ~9 million residents. The city of Tehran is divided into 22 municipal districts. There are approximately four million vehicles in this city, with millions of liters of fuel used and the presence of polluting industries like petrochemicals or natural gas refineries, heat power plants, etc., making Tehran one of the largest polluted cities in Iran^14^. The severity of the air pollutants has resulted in several school closures and increased respiratory-related hospital admissions^15^.

### Cancer data

In order to investigate the effects of land use on the incidence of lymphoma and leukemia cancer, a hospital-based case–control study was employed. For this purpose, data on children diagnosed with leukemia and lymphoma between 2017 and 2021 was gathered. Additionally, in order to provide comprehensive coverage of various regions within Tehran, three Children's Hospital Medical Centers, namely Ali Asghar, Mofid, and Imam Khomeini, were selected as the primary data collection centers. Both the control and case groups were composed of individuals aged between 1 and 15 years. The control group was subsequently chosen from the same hospitals as the cases to ensure socioeconomic similarity. Controls were randomly chosen and consisted of healthy children undergoing regular check-ups or visiting the pediatric clinic for non-blood-related issues such as fractures, osteoarticular disorders, trauma, injuries, gastrointestinal diseases, infections, and poisoning. Eligibility was limited to individuals who had resided in the same home for a minimum of one year before the diagnosis date and were under the age of 15 at the time of diagnosis. Exclusion criteria were applied to maintain the quality of the data. These criteria included children with Down syndrome, those whose geographic address was not recorded in their file, and those whose individual-level information was incomplete. The following childhood cancers were classified and identified using the International Classification of Childhood Cancer, 3rd Edition (ICCC-3)^16^: non-Hodgkin lymphoma (NHL; codes 022-023), acute myeloid leukemia (AML; code 012), Hodgkin lymphoma (HL; code 021), and acute lymphoid leukemia (ALL; code 011). Finally, to evaluate the exposure levels both for cases and controls, residential addresses for cases and controls were extracted from the patient's records. Subsequently, these addresses containing 10-digit postal codes were obtained from the Bureau of Iran's Post Office and geocoded using ArcGIS software and Google Earth.

### Covariates

We took into account possible risk factors associated with childhood cancers by utilizing data available in the patient's records for necessary adjustments. These factors contained details such as parental smoking status, the child's sex (male or female), birth year, and family history (a family history of cancer is a record of cancer information about a child and his or her close relatives).

### Land use exposure assessment

**Figure 1:**
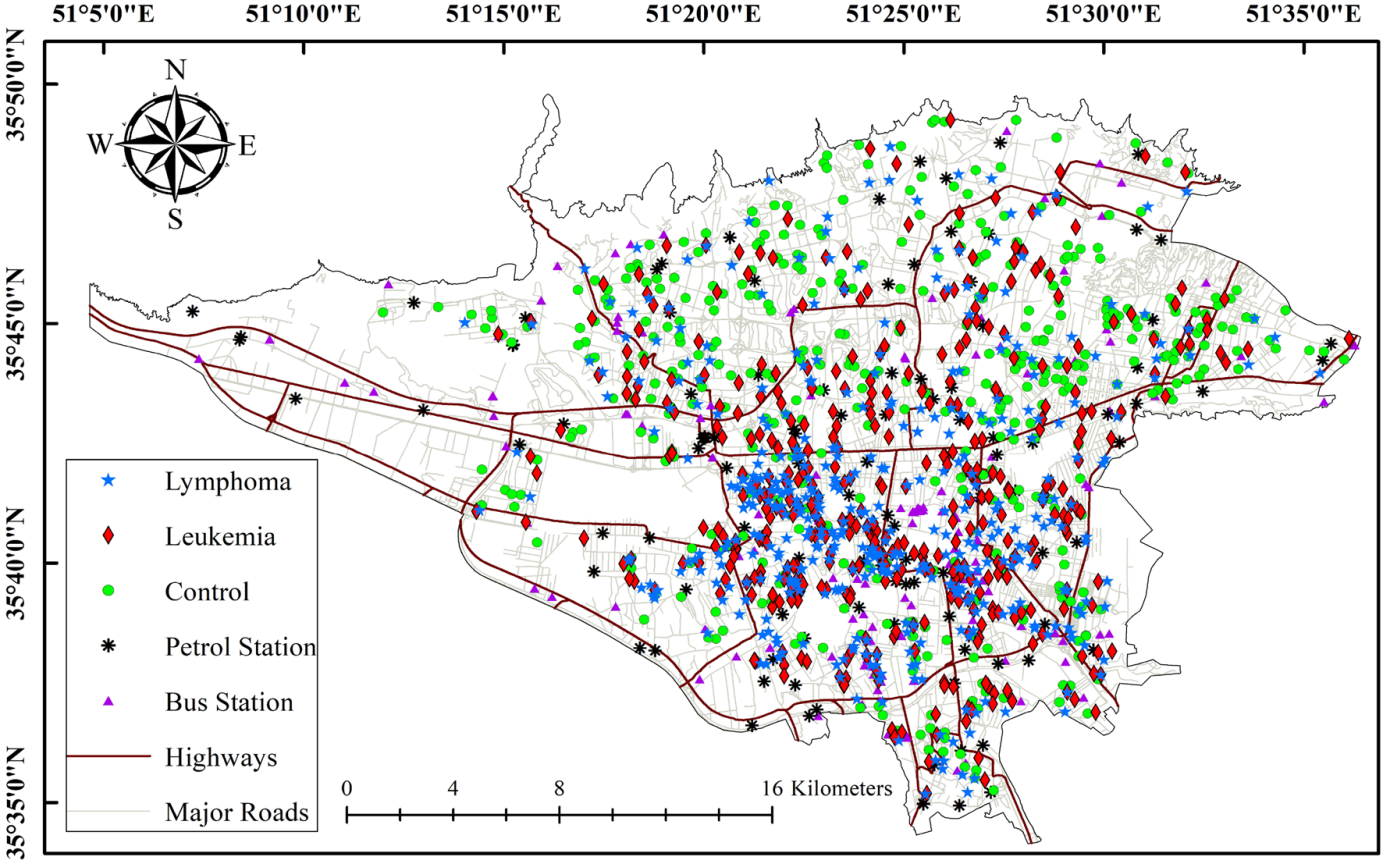
A map of Tehran city, depicting the geographic distribution of both cases and controls, petrol and bus stations, and roads/highways generated in the ArcGIS 10.8 software (http://www.esri.com).

A GIS-based method was used for exposure assessment to land use. In this method, circular buffers with different radii were created around different land use locations to calculate and assess the impact of each land use. The land use types investigated in this study included main roads, petrol stations, bus stations, and power lines. We focused on main roads, which were divided into Class 1 and 2 roads. Roads classified as Class 1, which typically refer to highways, are major roadways designed for heavy and high-speed traffic. They often contain two lanes or more in each direction, restricted access, and distinct carriageways for cars going in opposing directions. Class 2 roads are those constructed inside cities to handle heavy traffic flow. A class 2 road provides access to the highways and typically has at least one lane in each direction. They often feature stoplights, intersections, and roundabouts. We used different distance thresholds to determine the exposure between roads and the case and control groups. Distance thresholds of 0–100, 300, 500, and > 1000 m were utilized for highways, while for Class 2 roads, distance thresholds were 0–50 and 100 m. For power lines, we used two buffers with a radius of 0–50 m and 100 m, and two distances of less than 500 m and greater than 500 m were considered for bus stations. Petrol station exposure assessment involved considering distance thresholds of 100 m and 500 m. For each of the land use types under investigation, buffers with the specified distances were drawn in a separate GIS layer, and the locations of the cases and controls were determined within each layer. Ultimately, the number of cases and controls located within the buffers was determined. These calculations were performed using ArcGIS 10.8 (ESRI, Redlands, CA). An example of buffers around land use types is shown in Fig. 1. Statistical analyses required for determining the relationship between land use and the chance of childhood cancer were performed using the data obtained in this section.

### Statistical analyses

We used logistic regression to estimate the odds ratio (OR) and its associated 95% confidence interval (CI) to examine the association between land use and the incidence of lymphoma and leukemia among children. Based on data availability and existing research on putative risk factors for leukemia and lymphoma, we identified potential confounders. Then, to identify potential risk factors for childhood cancer, we employed a multivariate logistic regression model. Potential confounders of childhood leukemia and lymphoma included age (1–5, 5–10, 10–15 years), child sex (male or female), parental smoking status (yes or no), and family history (yes or no). Each of the potential risk factors was entered separately into the model, and then appropriate covariates were selected based on related studies or having a significant association (P < 0.20) with the risk of leukemia and lymphoma in our research. The Stata software version 17.0, developed by StataCorp, has been used to perform the statistical analysis.

### Ethical considerations

In accordance with the guidelines set forth by the institutional review boards and Research Ethics Committees of Ali Asghar, Imam Khomeini, and Mofid hospitals, as well as the Research Ethics Committees of Iran University of Medical Sciences (IR.IUMS.REC.1400.1078), informed consent was deemed unnecessary for our study. This waiver was granted due to the exclusive reliance on data extracted from patients' records and the absence of direct human experimentation or tissue sampling. Nonetheless, ethical considerations remained paramount, and the research was performed following relevant guidelines and regulations.

## Results and discussion

The primary analysis encompassed a total of 428 cases of leukemia, 428 cases of lymphoma cancer, and 428 control subjects. The demographic characteristics of the participants are shown in Table 1. Additionally, the distribution of covariates for both cases and controls is presented in Table 1. Among the 428 leukemia patients that were in the study, 328 were diagnosed with ALL, and 100 were diagnosed with AML. Likewise, within the lymphoma cases, 97 were attributed to NHL and 331 to HL. The general demographics for childhood leukemia and lymphoma yielded approximately similar results. In order to provide a full overview, Fig. 1 illustrates the geographic distribution of the residential locations of individuals within both the case and control groups. Both cases and controls exhibited similar distributions in terms of age, sex, family history, and parental smoking.

**Table 1:** Distribution of possible risk factors of cases and controls associated with Leukemia and Lymphoma childhood cancer (diagnosed in 2017–2021).

Characteristic	Values	Leukemia (n = 428) No. (%)	Lymphoma (n = 428) No. (%)	Controls (n = 428) No. (%)
Sex	Male	60.75	58.41	57.01
	Female	39.25	41.59	42.99
Age group distribution	1–5 years	42.75	42.52	42.75
	5–10 years	41.58	40.89	41.12
	10–15 years	15.65	16.59	16.12
Family history	Yes	10.98	12.62	11.92
	No	89.02	87.38	88.08
Parental smoking	Yes	25.23	26.17	27.10
	No	68.46	69.39	68.45
	Missing	6.31	4.44	4.43

### Exposure to the main roads

In this study, we looked into the association between a child's risk of developing leukemia or lymphoma and their closeness to a highway. Our focus was on main roads, which include highways and Class 2 roads. The measurement criterion for exposure assessment was the buffer around the main roads. These buffers varied from 100 to 1000 m for highways, and we used distances of 50 and 100 m for Class 2 roads. These distances indicated the proximity between the roads and the case or control groups. For highways, individuals living < 100 m from a highway had the highest exposure to pollutants. In contrast, the assumption is that children who lived > 1000 m from the major road were least exposed. These predefined distances are based on studies that have modeled the dispersion of pollutants from roads^17^. Table 2 presents the findings from this investigation.

**Table 2:** Associations between exposure to different types of land use (residence near highways and 2 roads, petrol stations, bus stations, and power lines) from the time of birth to the diagnosis date and risk of Leukemia and Lymphoma childhood cancer in Tehran 2016–2021.

Leukemia	Values	OR(95%CI)	
Possible risk factors		Crude	Adjusted^a^
Sex	Male/female	1.16 (0.88–1.53)	1.17 (0.89–1.53)
Age group distribution	Years	1.06 (0.79–1.16)	1.00 (0.96–1.04)
Family history	Yes/no	0.91 (0.59–1.38)	0.91 (0.59–1.40)
Parental smoking	Yes/no	0.94 (0.76–1.15)	0.94 (0.76–1.16)

Types of land use	Distances	Crude	Adjusted
Highways	100 m	*1.87 (1.00–3.49)*^b^	*1.82 (0.97–3.42)*
	300 m	*1.46 (1.03–2.06)*	*1.42 (1.01–2.02)*
	500 m	*1.35 (1.00–1.82)*	*1.34 (0.99–1.80)*
	1000 m	0.78 (0.59–1.02)	0.78 (0.60–1.03)
Roads class 2	50 m	1.05 (0.79–1.39)	1.04 (0.78–1.39)
	100 m	0.96 (0.71–1.30)	0.96 (0.71–1.30)
Petrol station	100 m	*2.15 (1.00–4.63)*	*2.12 (0.98–4.58)*
	500 m	1.41 (0.91–2.20)	1.44 (0.92–2.24)
Power line	50 m	*3.05 (0.97–9.55)*	*2.90 (0.92–9.14)*
	100 m	0.94 (0.47–1.85)	0.96 (0.48–1.90)
Bus station	500 m	1.29 (0.87–1.90)	1.34 (0.90–1.98)
	> 500 m	1.19 (0.91–1.57)	1.15 (0.88–1.52)

Lymphoma	Values	Crude	Adjusted^a^
Possible risk factors			
Sex	Male/female	1.05 (0.80–1.38)	1.07 (0.81–1.40)
Age group distribution	Years	1.01 (0.84–1.22)	0.93 (0.90–0.97)
Family history	Yes/no	1.06 (0.70–1.60)	1.09 (0.72–1.65)
Parental smoking	Yes/no	0.98 (0.81–1.19)	1.01 (0.84–1.23)

Types of land use	Distances		
Highways	100 m	*1.71 (1.00–2.93)*	*1.71 (1.00–2.92)*
	300 m	1.22 (0.86–1.72)	1.11 (0.77–1.59)
	500 m	1.25 (0.93–1.69)	1.18 (0.87–1.61)
	1000 m	1.23 (0.94–1.61)	1.19 (0.90–1.56)
Roads class 2	50 m	1.01 (0.76–1.34)	1.01 (0.76–1.34)
	100 m	1.11(0.82–1.51)	1.13(0.83–1.53)
Petrol station	100 m	1.09 (0.47–2.50)	1.05 (0.46–2.43)
	500 m	1.02 (0.64–1.64)	1.01 (0.63–1.62)
Power line	50 m	1.50 (0.24–9.04)	1.56 (0.25–9.39)
	100 m	0.71 (0.41–1.22)	0.67 (0.38–1.16)
Bus station	500 m	1.40 (0.95–2.06)	1.38 (0.93–2.03)
	> 500 m	1.28 (0.97–1.67)	1.27 (0.97–1.67)

^a^Adjusted for highways, roads class 2, petrol station.

^b^Italicized values indicate statistical significance at p < 0.05.

The results of this study indicate that the incidence of childhood leukemia is positively correlated with residing 100 m or less from a roadway (OR = 1.87; 95% CI 1.00–3.49).These results indicate that children residing within 100 m of highways have a 1.87 times higher chance of developing leukemia compared to children living at a distance of 1000 m. Furthermore, the data for Class 2 roads suggest that there is no statistically significant association between children who live within 50 m of Class 2 roads and the chance of having leukemia (*p*-value: 0.717). The findings also indicated a significant relationship between the incidence of lymphoma in children and the residence's closeness to highways (< 100 m) (OR = 1.71; 95% CI 1.00–2.93) (Table 2). However, for Class 2 roads, no significant association was observed (*p*-value: 0.942).

Living close to major roads and highways was linked to a higher risk of cancer in children, according to the findings of a Swiss cohort study^18^. In a meta-analysis study that used traffic density as an exposure parameter, the pooled OR was 1.09. When only studies of ALL were examined, the OR increased to 1.26^19^. In 2014, a meta-analysis conducted by Boothe et al. examined fifteen different studies. Their results showed that exposure to traffic during the postnatal period was associated with leukemia in children (OR: 1.53; 95% CI = 1.12–2.10), but no association was observed during the pregnant period (OR: 0.92; 95% CI = 0.78–1.09)^20^.

In general, living close to busy highways may be associated with an increased risk of children's cancer, according to the data, although not all studies have found this association^21^. In several other studies that have addressed this topic, the difference in results could be attributed to differences in exposure classification. The observed variations in results across distinct research could be partly attributed to the disparate definitions of exposure^19^. In the present study, the obtained ORs are larger than the ORs presented in the meta-analyses, but they are consistent with the results obtained from some other studies. As an example, a study carried out in the United States revealed an OR of 2.70^22^, while a study done in the United Kingdom indicated an OR of 1.61^23^.

Despite the available research, the exact mechanism of the association between living near roads and childhood cancer is not fully understood. This relationship may be due to the simultaneous presence of several potential factors, since high-traffic roads are an important source of air pollution. Particulate matter (PM), carbon monoxide (CO), nitrogen oxides (NOx), ultrafine particles (UFP), black carbon (BC), and polycyclic aromatic hydrocarbons (PAHs) are some of the pollutants that fall under this category^24^. There is an association between these pollutants and a higher risk of pediatric cancer^25,26^. Living near highways and major roads can expose people to higher levels of pollutants emitted from vehicles. Numerous studies demonstrate that PM exposure varies geographically within cities, and more detailed spatial analysis indicates that residents of areas with heavy traffic are at increased risk^27^. Because residing in these polluted zones might have detrimental impacts on one's health due to the harsh gradients of pollutants near roadways^24^. According to the findings of Filigrana et al., there was an increase in PM_2.5_ and NO_2_ concentrations close to roadways. In their study, the concentrations of NO_x_ and PM_2.5_ near highways (300 m) were 30 ppb and 1.8 µg/m^3^, respectively^28^. In Amsterdam, Fischer et al.^29^ examined the concentrations of indoor VOCs, PPAH, PM_2.5_, and PM_10_, as well as those outside, of residences close to high- and low-traffic volume streets. They found that PPAH and VOC levels were about twice as high in high-traffic indoor and outdoor areas as they were in low-traffic regions^29^. Previous studies have indicated that the areas around roads have higher concentrations of BC, NOx, UFP, and CO. It is believed that these pollutants can enter the body through inhalation and cause genetic mutations or damage to the immune system. These alterations may result in the development of cancer^30^. In a study, the damage caused by UFP on DNA and its relation to cancer were investigated. This study assessed the personal exposure of participants to UFP, DNA oxidation, and strand breakage in mononuclear blood cells while they rode bicycles through Copenhagen traffic. Cycling in traffic increased exposure to UFPs and DNA oxidative damage^31^. Consequently, living near highways exposes people to a complex mix of harmful pollutants that can have significant health consequences, especially for vulnerable populations, especially children.

### Exposure to the petrol station

In Tehran, there are a total of 123 petrol stations (Fig. 1). Due to the emission of gasoline vapor, petrol stations are natural sources of BTEX in urban areas. As a result, it is anticipated that as petrol stations get closer, the amount of BTEX in the air will rise^47^. According to the results of studies, these pollutants increase the risk of cancer^48^. However, no research has looked at the relationship between the incidence of childhood cancer and the distance of a dwelling from a petrol station in Tehran up to this point. Therefore, in this study, considering the exact location of petrol stations, we calculated exposure risks based on buffer thresholds of 100 m and 500 m, and Table 2 summarizes the related results. The results indicated that children who lived less than 100 m from a petrol station had a higher OR for leukemia (2.15) than children who lived 500 m or farther away. These findings suggest an association between residing close to a petrol station and the incidence of leukemia. Nevertheless, there was no statistically significant association discovered between lymphoma and residing within a 100-m radius of a petrol station. As shown in Table 2, the results did not change after considering the confounding factors (OR: 1.05; 95% CI 0.46–2.43). Based on the obtained results, this relationship seems less clear for lymphoma compared to leukemia. As far as the authors know, before our study, no research had looked into the relationship between childhood lymphoma and living close to a petrol station. The results of the current study show this association is less evident for lymphoma in comparison to Leukemia. Much research has looked into children who are exposed to benzene and their chance of developing lymphoma, though the evidence for this cancer is not as strong^49,50^.

Living next to a petrol station can be considered a risk factor for childhood cancer, given the rise in the incidence of cancer over the past three decades and the findings of this study. Petrol stations can indeed contribute to air pollution as a result of activities such as fuel refueling and petrol leaks. Pollutants released into the atmosphere and fuel leaks are possible outcomes of these activities^35^. When handling and storing gasoline at petrol stations, volatile organic compounds (VOCs) like benzene and 1,3-butadiene may be emitted^35^. These substances are recognized as air pollutants, and when humans are exposed to them, they can be harmful to human health. The average values of total VOCs, benzene, toluene, and xylene were reported to be 647.01, 161.22, 200.81, and 229 ppm, respectively, in a survey that was done in six petrol stations in Tehran^51^. These results exceeded the World Health Organization's (WHO) recommended levels of outdoor ambient air. Studies have associated exposure to BTEX compounds at the environmental level with health effects such as reduced fetal growth, cardiovascular diseases, sperm abnormalities, and respiratory problems^52^. Specifically, benzene exposure has been connected to spontaneous abortions, activation of oxidative stress pathways, abnormal sperm production, and decreased immune cells and antibodies^53^. It is significant to note that in 1987, the International Agency for Research on Cancer (IARC) designated benzene as a class 1 carcinogen^54^. Toluene also causes changes in the reduction of liver and kidney function, the activation of oxidative stress pathways, and the number of immune cells^55^. Exposure to ethylbenzene had a relationship with elevated markers of oxidative stress, while exposure to xylene was linked to an increased incidence of oligomenorrhea^56^. Research has indicated that the evaporation of gasoline causes a higher concentration of xylene and ethylbenzene in the air surrounding petrol stations^47^. It is important to remember that prolonged exposure to ethylbenzene may raise the chance of developing cancer^47^. As a result, the high concentration of these pollutants in the vicinity of petrol stations in Tehran's metropolis can, therefore, severely compromise the health of locals.

The findings from earlier research are collected in Table 3. Research has indicated that the discharge of benzene and other contaminants from petrol stations has been associated with a higher incidence of leukemia in children^13^. Nonetheless, a few studies have found an insignificant association between children who live close to petrol stations and an increased incidence of leukemia^57^. This difference in findings could be attributed to differences in the exposure considerations in studies, such as different urban contexts among cities and/or the type of fuel distributed at petrol stations. Moreover, some studies recorded exposure to petrol stations through maternal interviews, which may cause recall biases^32^. We used the distance between the residence and petrol stations as the exposure factor.

**Table 3:** A summary of multiple case–control studies that looked into the potential association between the risk of developing childhood cancer and the distance of children's homes from different land uses (such as petrol stations, highways, road class 2, power lines, and bus stations).

Ref.	Region	Type of data collection	Outcome	Age	Exposure			
					Type land use	Contrast	OR (95% CI)	Cases
^32^	France	Population	ALL and AML	0–18	Petrol stations	Yes/no	2.1(1.1 to 4.0)	765
^23^	UK	Population	Leukemia	0–15	Main roads Petrol stations	< 100 m	Main roads:1.16 (0.74 to 1.72) Petrol stations: 1.99 (0.73 to 5.43)	–
^33^	France	Hospital	Leukemia	0–14	Main roads Petrol stations	Petrol stations: yes/no Road:50 m	Leukemia: 4.0 (1.5 to 10.3) ANLL: 7.7 (1.7 to 34.3) All road types: 0.9 (0.7 to 1.3)	285
^34^	Switzerland	Population	All cancers	< 16	Petrol stations	< 50 m	All cancers: 1.29 (0.84–1.98) Leukemia: 1.08 (0.46–2.51)CNS tumors:1.30 (0.51–3.35)	6129
^35^	Northern Italy	Population	Leukemia	0–14	Petrol stations	< 1000 m	2.2 (0.5–9.4)	182
^32,36^	Taiwan	Population	Leukemia	0–14	Petrol Station Density ^a^	N/km^2^	1.91 (1.29–2.82) for more density	729
^37^	California	Population	All cancers	< 5	Highway and major roads	500-ft (road density)	All cancer 0.87(0.75–1.00) Leukemia 1.0 (0.92 for both) CNS 1.22 (CI = 0.87–1.70)	1728
^38^	Italy	Population	Non-Hodgkin lymphoma	≤ 10	Main roads	100 m	1.00 (0.80 to 1.41)	620
^39^	Spain	Population	Leukemia	0–14	Roads	50 and 100 m	2.90 (1.30–6.49)	1061
^40^	Italy	Population	Leukemia	0–14	Major road	20–150 < 20 m	20–150 m: 1.95 (0.99–3.86) < 20 m: 2.09 (0.85–5.12)	120
^41^	French	Population	Leukemia	< 15	Major road	< 150 m	AML: 1.2 (1.0, 1.4) ALL: 1.0 (0.9, 1.1)	2760
^42^	Oklahoma	Population	Leukemia	< 20	roads	500 m and 750 m	500 m: 1.03 (0.79, 1.35) 750 m: 1.01 (0.78, 1.31)	
^26^	France	Population	Acute leukemia	< 15	Major road	< 500	Heavy-traffic: 2.0 (1.0–3.6) Density: 2.2 (1.1–4.2)	763
^13^	Northern Italian	Population	Leukemia	0–15	power lines	< 100 m	2.0 (95% CI 0.8–5.0)	182
^43^	Brazil	Hospital	ALL	0–14	power lines	50 m 200 m	50 m: 3.57 (0.41–31.44) 200 m: 1.67 ( 0.49–5.75)	162
^44^	California	Population	Leukemia CNS	< 16	power lines	< 50 m	Leukemia: 1.4 (0.7–2.7) CNS: no evidence	5788
^45^	England and Wales	Population	All cancers	0–14	power lines	200 m 600 m	Leukemia: 200 m: 1.69 (1.13 to 2.53) 200-600 m: 1.23 (1.02 to 1.49)	29,081
^46^	Sweden	Population	All cancers	< 16	Power lines	300 m	0.2 µT: 2.7 (1.0–6.3) 0.3 µT: 3.8 (1.4–9.3)	142
This study	Tehran	Hospital	Leukemia	1–15	Petrol stations Highway Road Bus station Power lines	ps = 100,500 m h = 100,300,500,1000 r = 50,100 bs = < 500, > 500 pl = 50,100	Ps (100 m): 2.15 (1.00–4.63) h (100 m): 1.87 (1.00–3.49) pl (50 m): 3.05 (1.07–9.55 )	428
This study	Tehran	Hospital	Lymphoma	1–15	Petrol stations Highway Road Bus station Power lines	ps = 100, 500 m h = 100, 300, 500, 1000 r = 50, 100 bs = < 500, > 500 pl = 50, 100	H (100 m): 1.71 (1.00–2.93)	428

^a^Number of stations per km^2^.

According to the results obtained, the risk of leukemia in children decreased with increasing distance from petrol stations, and this relationship was not significant. These findings are similar to those of other studies. For example, researchers showed that outdoor BTEX concentrations were higher in zones around gas stations with distances up to 30–50 m compared to areas with distances of 60–90 m or 100 m^58^. In a study conducted in Tehran, it was discovered that the annual average levels of benzene and BTEX were higher within 300 m distance from petrol station locations than they were outside of this buffer zone, at 67.3 μg/m^3^ and 9.1 μg/m^3^, respectively^59^. Likewise, in a Brazilian investigation, ambient air samples taken close to petrol stations revealed concentrations of BTEX chemicals 2.4–3.7 times higher than samples taken at a distance of 250 m from station^60^. This significant difference indicates that the evaporative emission of BTEX compounds strongly influences the air around petrol stations. Given that children spend 90% of their time indoors, outdoor sources of BTEX, such as petrol stations, may increase interior pollution and expose children to additional risks^52^. The results of a study showed that the concentrations of BTEX both indoors and outdoors are often higher near gas stations than they are at further distances of 40 or 80 m^58^. Considering their proximity to the source of pollution, long-term exposure to BTEX compounds may thereby raise the risk of cancer. A significant prevalence of hematopoietic system and lungoid tissue Malignant neoplasms with exposure to concentrations exceeding the established levels of benzene has been observed within 75 m of petrol stations in Porto, Portugal^61^. Because benzene and other toxins are released into the air by petrol stations, living close to one can raise a child's risk of developing leukemia.

### Exposure to the bus stations

In recent years, due to industries moving away from urban areas, bus stations have become the dominant source of pollution^62^. Since buses are the only diesel vehicles that are driven often during the day, the majority of them have big diesel engines, which are significant sources of pollution in cities. With the increase in population and the activity of buses, the pollution of buses has been increasingly noticed and emphasized. There are few studies on bus emissions at bus stations. Based on research conducted, a strong association exists between childhood cancers and greenhouse gas emissions, especially in children living near bus stations^63,64^. Consequently, this study looked into the relationship between children's proximity to bus stations and the incidence of lymphoma and leukemia in Tehran's children. The results of this investigation are presented in Table 2. As noted, there is no association between leukemia and residing less than 500 m from a bus station (p-value: 0.20). Similarly, no significant association was observed for lymphoma cancer.

Researchers from the University of Birmingham showed that the risk of cancer-related mortality in children living within 500 m of bus stations is six times higher^65^. It is significant to remember that the association between residential proximity to bus stations and cancer risk may result from bus emissions rather than the bus station itself^65^. Studies have used a variety of techniques to calculate the pollutant concentrations at bus stations and have shown that PM_10_ and PM_2.5_ concentrations are significantly higher than the established air quality criteria for America, Europe, and Asia^66,67^. For example, Cevallos et al. found that PM_2.5_ concentrations ranged from 13.66 to 25.72 µg/m^3^ at seven bus stops on the University of Manchester campus^68^. In another study, Xu et al. discovered that PM_10_ was 2.5 times greater in bus stations in China during rush hour (254 ± 128 μg/m^3^) than it was 24 h later (103 μg/m^3^)^69^. Based on estimations, PM_1_, PM_2.5_, and PM_10_ comprise 89% to 94% of all exhaust particles and are the most common types of exhaust particles observed near bus stations^67^. On the other hand, bus stations that are close to roads can accumulate large amounts of particulates, resulting in high levels of exposure to direct vehicular emissions^62^. Pollutants emitted by vehicles, including buses, have been identified as potential carcinogens^70^. Moreover, the study by Shuang Li et al. investigated air pollution produced by buses and bus stations in China, and according to their findings, there was a significant amount of monoaromatic hydrocarbons (MAHCs) in the air close to bus stations and buses themselves^71^. However, there is no specific study that directly examined the proximity of residences to bus stations and its association with childhood cancer. Therefore, more research must be carried out into the potential impact of air pollution from bus stations on cancer risk in children.

### Exposure to the power line

It's still unclear how living close to power lines affects health; one such effect is childhood leukemia. According to the IARC, magnetic fields greater than 0.4 μT may cause human cancer (Group 2B), particularly children's leukemia^72^. Previous research has revealed that over 1.6 million people, or 20% of Tehran's total population, live close to high-voltage power lines that carry 123, 230, and 400 kilovolts^73^. This is the reason we looked into the relationship between proximity to power lines and the risk of children's lymphoma and leukemia. The results of this investigation are presented in Table 2, considering two distances: 50 and 100 m. Results pertaining to leukemia indicated that children residing within 50 m of power lines had a greater chance of developing leukemia (OR 3.05; CI 0.97–9.55). It's crucial to remember, though, that this evaluation was based on a very limited sample—just 12 cases. According to the results obtained in Table 2, no association was observed with increasing distances up to 100 m from power lines. It is clear from looking at the statistics in the table that there is no association between the incidence of lymphoma and residing less than 50 m from power lines. Similarly, a study in Sweden found no association between children's incidence of lymphoma and their proximity to power lines^44^.

Numerous research studies have looked into the association between children's cancer risk and a home's closeness to power lines (Table 3). For example, case–control research was carried out in the UK by Draper et al. According to their research, there is an association between childhood leukemia and the distance between a residential address and the closest high-voltage power line^74^. Combining data from six separate studies, Crespi et al. concluded that there was an association between the distance between the home and power lines of different voltages and the incidence of childhood leukemia^44^. They recorded an OR of 1.6, 1.3, or 1.2 for distances of 0 to 50 m, 50–100 m, and 100–200 m from the closest power line, respectively^44^. A meta-analysis of 11 published case–control studies, however, revealed no association between childhood leukemia and the proximity of the closest overhead power line. The adjusted OR for leukemia in children who lived less than 50 m away from power lines that had a higher voltage (200 kV or more) was 1.33 (95% CI 0.92–1.93)^75^.

In conclusion, our research showed that children living 50 m away from power lines had a slightly higher risk of developing leukemia than children living 100 m away. Research on the association between exposure to magnetic fields produced by power lines and the incidence of cancer in children is still underway. Research has yielded varying conclusions, some of which point to a possible association between children's exposure to magnetic fields of power lines, especially when those distances are shorter, and an elevated risk of cancer. However, this association has often been observed in small sample sizes. It is noteworthy that challenges related to the characteristics of each study site, such as population mobility and changing addresses, have the potential to bias. For this reason, the explanation of this relationship seems complicated. As a result, it's essential to consider the limitations of small sample sizes and the potential influence of confounding factors. More research is required to fully comprehend the possible health implications of being exposed to magnetic fields from power lines.

## Limitations

In this study, individual data that may confound this association, such as lifestyle or socio-economic variables, were not examined due to their unavailability in the study. In this study, we considered the distance of land use type as a proxy for pollution source and an indicator of exposure. However, actual exposure significantly depends on factors such as wind patterns and geographical features. Despite this limitation, this study uses distance classification highways, Class 2 roads, petrol stations, bus stations, and power lines, which can help reduce the limitation. Nevertheless, it is important to note that this limitation may challenge the ability to detect positive results but does not undermine the validity of the observed associations. On the other hand, using distance measures from roads and highways or petrol stations and bus stations does not allow for assessing effects on particular toxins; they can be regarded as a limitation. However, instead of being exposed to specific compounds, people are exposed to a variety of air pollutants in reality. During our investigations in Tehran, we encountered specific challenges. These challenges could have created biases in our findings. One of the noteworthy points is that families often move from different regions of Iran to hospitals or the city of Tehran when their children are diagnosed with cancer. However, we did not include children who changed their residence after diagnosis in our study.

## Conclusion

In this research, we looked into any potential associations between the risk of childhood cancer, particularly leukemia and lymphoma, and residential proximity to different land use types, such as petrol stations, major roadways, bus stations, and power lines. Findings showed significant correlations, as the OR for children with leukemia and lymphoma within 100 m of highways were 1.82 and 1.71, respectively, based on logistic regression adjusted for confounding factors. In addition, the OR for leukemia was 2.15 when residing within 100 m of a petrol station. No association was found between lymphoma and being close to petrol stations. According to the study's findings, there is no correlation between a child's residence and the bus station for those who have lymphoma or leukemia. On the other hand, our findings showed that children who lived 50 m or less closer to power lines were at greater risk of getting leukemia. Living close to highways and petrol stations may increase the risk of childhood leukemia and lymphoma, according to this study. However, more studies are needed to obtain better results by considering other individual-level data.

## Data Availability

The datasets utilized and/or reviewed in this study are not available to the public due to data-sharing regulations mandated by Ali Asghar, Imam Khomeini, and Mofid hospitals. No data generation or analysis is reported in this manuscript.
